# Ticks and fleas of the critically endangered mountain pygmy-possum (*Burramys parvus*) in Kosciuszko National Park, Australia

**DOI:** 10.1016/j.ijppaw.2026.101232

**Published:** 2026-04-17

**Authors:** Danielle J. Oste, Damien P. Higgins, Hayley Bates, Linda Broome, Floris F. van Ogtrop, Mingeun Cho, Stephen C. Barker, Jan Šlapeta

**Affiliations:** a Sydney School of Veterinary Science, Faculty of Science, The University of Sydney, Sydney, NSW, Australia; b School of Medical Sciences, Faculty of Medicine and Health, The University of Sydney, Sydney, NSW, Australia; c Biological, Earth and Environmental Sciences, University of New South Wales, Kensington, NSW, Australia; d Conservation Programs Division, Department of Climate Change, Energy, the Environment and Water, PO Box 733, Queanbeyan, NSW, Australia; e School of Life and Environmental Sciences, Faculty of Science, The University of Sydney, Sydney, NSW, Australia; f Department of Parasitology, School of Chemistry and Molecular Biosciences, The University of Queensland, Brisbane, QLD, Australia; g Sydney Infectious Diseases Institute, The University of Sydney, Sydney, NSW, Australia

**Keywords:** Mountain pygmy-possum, Ectoparasite, Wildlife conservation, Arthropod, *cox*1, Mitogenome

## Abstract

The mountain pygmy-possum (*Burramys parvus* Broom, 1896) is a critically endangered marsupial, native to alpine-subalpine regions of Australia. Little research has been conducted on their health, including no formal parasite surveys. Parasite dynamics can reflect host and ecosystem health, acting as sensitive indicators of changing environmental, social, intra- and inter-species interactions and can contribute directly to host species knowledge*.* Reports of parasites affecting *B. parvus* are limited, with most from incidental observations. This study sampled ticks and fleas from *B. parvus* at eight sites in Kosciuszko National Park, New South Wales, Australia. A combination of morphological and molecular techniques was used to identify specimens, and screen for *Rickettsia* and *Bartonella.* The ticks were identified as *Ixodes tasmani* Neumann, 1899 and *Ixodes* sp. cf. *tasmani* Neumann, 1899. *Ixodes heathi* Kwak, 2018 was not found. The ticks that underwent molecular identification based on partial *cox1* belonged to *I. tasmani* group within two distinct phylogenetic clusters*.* New mitogenomic reference sequences for two tick taxa and four flea species were obtained. Fleas were identified morphologically as *Acanthopsylla rothschildi* (Rainbow, 1905), *Acanthopsylla scintilla* (Rothschild, 1936), *Pygiopsylla hoplia* Jordan and Rothschild, 1922 and *Stephanocircus simsoni* Rothschild, 1905*.* Molecular screening found no evidence of *Bartonella,* one positive flea for *Rickettsia,* and a single suspect (late amplification) for *Rickettsia.* Statistically, greater *B. parvus* body weight was associated with decreased odds of tick presence and load, and male *B. parvus* had higher odds of flea presence. This study provides a systematic, standardised and formal survey of ticks and fleas of *B. parvus* in New South Wales, Australia, and establishes a baseline for future investigations into the impact of ectoparasites on the welfare of this critically endangered marsupial, supporting improved conservation measures across its restricted distribution.

## Highlights

• First baseline survey of parasites on the endangered mountain pygmy-possum.
• Identified tick and flea species using morphology and DNA methods.
• Very little *Rickettsia* DNA found and no *Bartonella* DNA detected.
• Smaller possums had more ticks; males had more fleas.
• Provides a reference point for future monitoring and conservation.

## Graphical abstract

**Figure:**
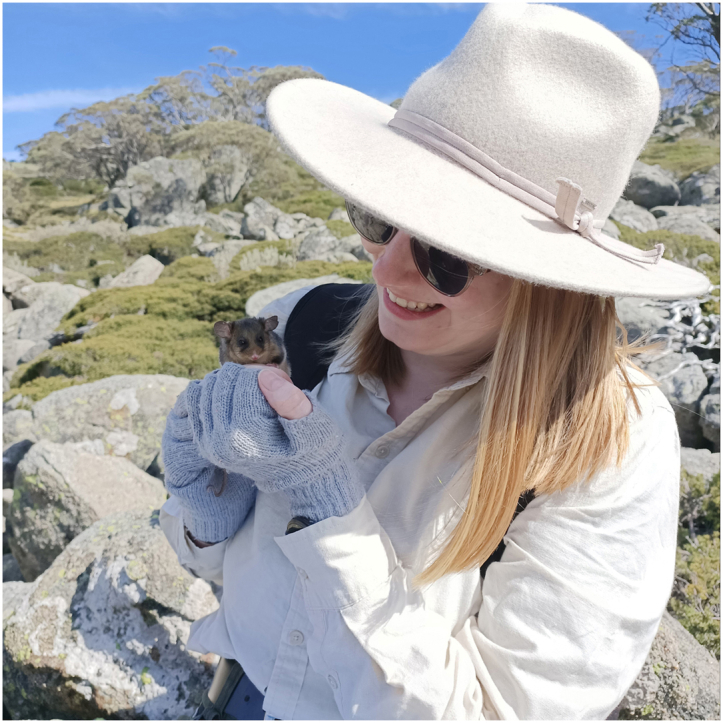

## 1 Introduction

**Fig. 1:**
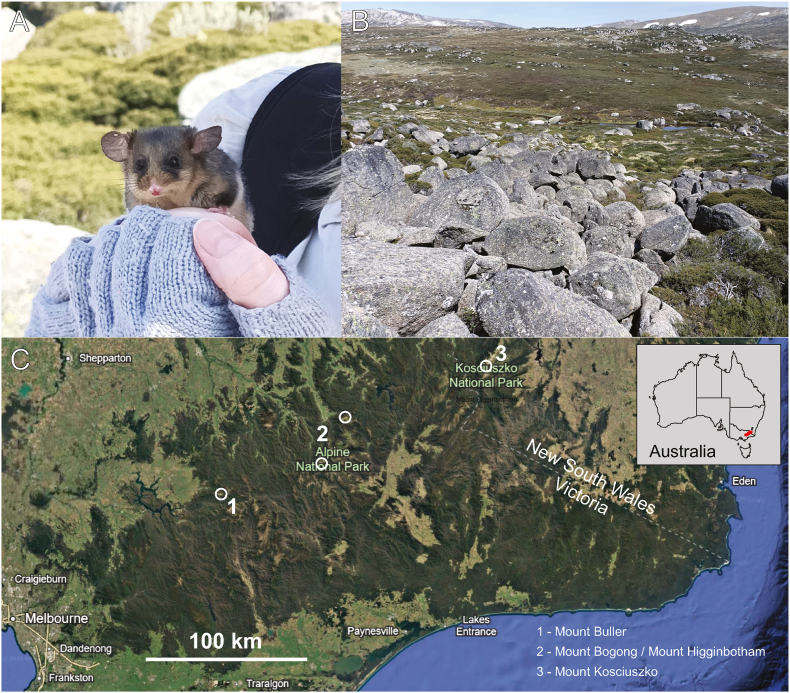
The critically endangered mountain pygmy-possum (*Burramys parvus*) from south-east Australia. A) The possum is a small, mouse-sized nocturnal marsupial. B) Its biotope is alpine boulderfields, Kosciuszko National Park shown. C) The distribution consists of three discrete populations (1-3). Our study site was at locality 3 in New South Wales, Australia. This map includes data from: Google, Landsat/Copernicus, SIO, NOAA, U.S. Navy, NGA, GEBCO, LDEO-Columbia, NSF, NOAA. Imagery from the dates: 1/1/2021. Map courtesy of Google Maps. Inset shows Australia with the location of the relic populations of *B. parvus* in red.

*Burramys parvus* (mountain pygmy-possum) is a critically endangered, terrestrial marsupial, native to alpine-subalpine regions of Australia (Menkhorst et al., 2008). They exist in three geographically isolated and genetically distinct populations, located close to the adjoining border of the two states of Victoria (Vic) and New South Wales (NSW) (Fig. 1) (Mitrovski et al., 2007). *Burramys parvus* live in periglacial blockfields and blockstreams known as ‘boulderfields’ (Heinze et al., 2004; Broome et al., 2013) and require a permanent water source (Bates, 2017). Other small mammals within their habitat include *Rattus fuscipes* (Waterhouse, 1839) (Australian bush rat), *Antechinus mimetes* (Thomas, 1924) (dusky antechinus), *Antechinus agilis* Dickman et al., 1998 (agile antechinus) and *Mastacomys fuscus* Thomas, 1882 (broad-toothed mouse) (Broome et al., 2013). Unlike arboreal possums, *B. parvus* nest beneath boulderfields and hibernate during winter, where snow provides thermal buffering (Heinze et al., 2004; Shi et al., 2015). Hibernation consists of repeated torpor bouts of lowered metabolic, physical and mental activity and body temperatures reduced from 37 °C to 2-3 °C, interspersed with brief arousals (Geiser and Broome, 1991; Broome and Geiser, 1995; Körtner and Geiser, 1998). After emerging in October, individuals gain weight and then mate; and following a two week gestation period, females give birth to supernumerary offspring, of which four attach to teats in the pouch, becoming nestlings at around 4 g at 30-45 days old (Mansergh and Scotts, 1990). *Burramys parvus* consume arthropods, seeds, and fruit, varying their diet to suit availability, season, and elevation (Gibson et al., 2018; Hawke et al., 2019). This species is considered to be at severe risk of extinction due to climate change, human activity, predation by invasive species, low genetic diversity and, habitat destruction and fragmentation (Mitrovski et al., 2007; Archer et al., 2019).

There is limited research on factors influencing *B. parvus* health*,* including parasite studies, such as surveys of common ectoparasites – ticks and fleas. *Ixodes tasmani* Neumann, 1899 species group ticks, are widespread generalist ticks with a very large host range that has been reported from 7 *B. parvus* in historical records (Barker and Barker, 2023). The recently discovered *Ixodes heathi* Kwak, 2018, believed to be a host specific, specialist tick, has been reported on *B. parvus* in populations from Victoria (Kwak et al., 2018). No adult *I. heathi* have been found, nor have there been reports on other hosts, and it is unclear if NSW *B. parvus* populations were surveyed (Kwak et al., 2018). A marsupial flea, *Acanthopsylla rothschildi rothschildi* (Rainbow, 1905) is widely distributed around eastern Australia and has been reported on *B. parvus* from NSW and there is a single record of an additional flea species, *Stephanocircus simsoni* Rothschild, 1905, on *B. parvus* from Victoria (Dunnet and Mardon, 1974). *Stephanocircus simsoni* is a less widely distributed flea, being found in Victoria, Tasmania and the Australian Capital Territory (Dunnet and Mardon, 1974).

Ectoparasites, particularly those with a wide host range, can transmit vector-borne bacterial infectious agents, and a wide diversity of *Bartonella* and *Rickettsia* species have been detected in *Ixodes, Acanthopsylla* and *Stephanocircus* species from Australian marsupials (Kaewmongkol et al., 2011; Ghafar et al., 2023). There are three main groups of *Rickettsia,* the spotted fever group, typhus group, and ancestral group (composed only of *Rickettsia bellii*) (Stenos et al., 2005). Both *Rickettsia* and *Bartonella* are widely distributed across the world and have One Health significance as many are zoonotic and pathogenic to humans (Ghafar et al., 2023). Depending on the level of interactions between small mammal species, ectoparasites and their associated infectious agents have the potential to spillover and spillback between species in the ecosystem, and this could potentially influence parasite dynamics of *B. parvus* (Kelly et al., 2009). Some of these infectious agents may be pathogenic, and ectoparasites can act as reservoirs and vectors for transmission to wildlife, or contribute to spillover events to humans (Ghafar et al., 2023). It is unclear what ticks and fleas affect *B. parvus* and if *Rickettsia* and/or *Bartonella* are circulating through these populations, nor is it known what factors influence ectoparasite dynamics in this ecosystem.

Parasite studies can inform researchers of species behaviour, nesting conditions, intra- and inter-species relationships, and environmental conditions. For example, a study on tammar wallabies found parasite burdens can be used to assess host body condition and be an indicator of habitat quality (Robert and Schwanz, 2013). A separate study, conducted on rodents living in high altitude sites, found parasite dynamics reflect and shift with changing environmental conditions (Gebrezgiher et al., 2023). Parasite dynamics have also been shown to reflect intra- and inter-species interactions, and a study completed on coastal environments found that parasites can be used as an indicator of community and ecosystem complexity (Moore et al., 2024). Such studies require a baseline, so researchers can understand the agents and host-parasite relationships present in a given time and space (Wood et al., 2026). This can then inform future study design and act as a reference point, against which to measure prevalence, population dynamics and environmental conditions within that ecosystem over time.

The aim of this study was to survey ticks and fleas of critically endangered *B. parvus* from the NSW population. To do this, data was recorded and samples collected from eight sites surveyed during the annual *B. parvus* monitoring survey of Kosciuszko National Park. A combination of traditional morphological and molecular techniques was used to identify ticks and flea samples and to screen for *Rickettsia* and *Bartonella* DNA. Host and habitat variables were analysed to identify any factors associated with tick and flea dynamics. This study provides the baseline for Kosciuszko National Park *B. parvus* populations, informing future monitoring and disease investigation, that may, in turn, inform research and conservation efforts.

## 2 Materials and methods

### 2.1 Sample collection

Ticks and fleas were collected during the annual monitoring survey of *B. parvus* populations located within Kosciuszko National Park, NSW, in October – November 2024, under ethics permit AEC 991129/01 (Department of Climate Change, Energy, the Environment and Water Animal Research Authority). *B*. *parvus* was trapped at eight (n = 8) sites across northern (Snow Ridge Hill, Rough Creek and Happy Jacks) and southern (Charlotte Pass, Whites River, Summit Road, Paralyser, Blue Cow) Kosciuszko (Table 1). Aluminium Elliot live-capture traps were lined with Dacron bedding, shielded from the rain with plastic bag covers, baited with crushed walnuts and set under boulders at known *B. parvus* sites. Between two and four traplines were set at each site, each with 20-50 traps, which were checked over four consecutive mornings.

**Table 1:** Sites where *Burramys parvus* was trapped and ectoparasites collected during annual monitoring survey in Kosciuszko National Park during October – November 2024. Aspect of boulderfields at site is the direction they face in relation to cardinal points.

Site	North or South	Mid-point Elevation^a^	Aspect of boulderfields at site
Charlotte Pass	South	1776	S
Whites River	South	1680	SSW
Summit Road	South	1918	NW
Paralyser	South	1805	bTrapline A + B: SW, C: WSW
Blue Cow	South	1867	S - SE
Snow Ridge Hill	North	1488	SW
Rough Creek	North	1612	NNE
Happy Jacks	North	1248	bTrapline HJ35: NE, HJ41: SE

a Metres above sea level.

b Traplines that have distinct aspects depending on the trapline used are noted here with their specific aspect. For the remaining sites, the traplines have the same aspect for all traplines used for this study.

Once trapped, *B. parvus* were weighed, scanned for a microchip, microchipped (if not captured previously) and examined visually to determine sex. Approximate age was recorded: adult (two or more years old); subadult (one year old) smaller in size, greyer pelage, and for females before young are born underdeveloped teats are visible in the pouch. Age approximation can be difficult based on visual assessment and so animals not clearly distinguished were classified as adult/subadult. Testis length was recorded for males, and reproductive details recorded for females by visual examination of pouch development, recording number and length of young if present, and condition/cleanliness of pouch and teats. Using a constant, equal effort*,* trapped *B. parvus* were examined visually to locate, record and collect parasites. *B*. *parvus* were examined visually for presence/absence of fleas (as direct counts could have resulted in inaccuracies as fleas quickly disperse during handling), by parting the fur over the entire animal and observing any fleas moving away from the light, or onto the handler, trap, or cotton handling bag, which were collected where possible. Each *B. parvus* was then examined visually by gently blowing apart the fur and using careful attention to look for ticks (adults, nymphs and larvae), beginning at the face/snout, with particular attention around the ears, then moving onto the body, followed by the legs and tail, then the underside and pouch using the same, systematic examination. A direct count was used to record the number and location of ticks observed (for example: ‘10 ticks right ear, 3 ticks head’). All ticks were removed where possible, using fine forceps to grasp the tick close to the skin, with gentle upward tension, to twist and pull the tick out in a slow, steady motion, taking care not to squeeze the tick body, or tear the tick. Forceps were cleaned between captures using a tissue to remove physical material, then passed through a flame to avoid cross-contamination of DNA. Not all parasites were collected, for example, if extreme weather required animals to be released from traps immediately, or, if not all parasites could be removed within the handling time approved by ethics permits. Ectoparasite samples were placed into 80% ethanol, with one vial per parasite type (tick or flea) for each *B. parvus*, and labelled with date, microchip identification, site, and trap number. Samples were stored at room temperature until analysis.

### 2.2 Morphological identification of tick and flea samples

Tick and flea samples were labelled with initials and then according to the number of tick or flea specimens collected from the individual possum (i.e. DOT1-2 for the second tick [T] taken from the first possum captured, DOF1-2 for the second flea [F] taken from first possum captured).

Ticks were examined by light microscopy (Olympus BX41 with Olympus DP21 digital microscopy camera) recording number of specimens, life stage (larvae, nymph or adult), condition (damaged or intact), and morphology. Tick morphological identification of adults was based on revised descriptions and keys for Australian ticks (Barker and Barker, 2023).

Identification based on flea morphology was conducted on all fleas using keys for Australian fleas (Dunnet and Mardon, 1974) by light microscopy (Olympus BX41 with Olympus DP21 digital microscopy camera). To enable clear visualisation for final morphological identification, fleas were cleared and mounted; fleas were soaked in 10% potassium hydroxide (KOH) at room temperature, until specimens were digested to visualise morphology. These samples were then dehydrated through a series of ethanol washes (70%, 80%, 90%, 96%) for 30 min each and mounted on a slide with Euparal (Cat. No. E295, Australian Entomological Supplies, South Murwillumbah, Australia) for final morphological identification.

### 2.3 Molecular identification of ticks and fleas

A random subset of tick samples from 17 *B. parvus* (containing 1-10 ticks each) from all 8 collection sites were subjected to DNA extraction and molecular identification as described previously (Huang et al., 2021). At least one flea from each of the *B. parvus* sampled (one to five fleas collected from 15 individuals) underwent DNA extraction, ensuring each genus (based on preliminary morphological identification) was assessed. Where there was more than one tick or flea collected, at least two were used. Fleas were incised on the dorsal caudal abdomen with a sterile scalpel blade and ticks were incised along the idiosoma, then placed into labelled Eppendorf tubes. The tubes were placed on a heat block at 60 °C to evaporate any remaining ethanol. Flea DNA was extracted using the Monarch Spin gDNA Extraction Kit (New England Biolabs, Australia) according to the manufacturer's animal tissue protocol and eluted gDNA stored at −20 °C. An extraction with no flea, and one with no tick, was included to serve as non-template controls (negative extraction control).

Once DNA was extracted, DNA samples underwent conventional PCR of partial cytochrome *c* oxidase subunit one (*cox1*). PCR amplification was performed in 25 μL using OneTaq® 2X Master Mix (New England Biolabs, Australia) and generic invertebrate primers (LCO1490: 5′-GGT CAA CAA ATC ATA AAG ATA TTG G-3′ and HCO2198: 5′-TAA ACT TCA GGG TGA CCA AAA AAT CA-3′) at 400 nM each, nuclease-free water and 2 μL of DNA, as described previously (Folmer et al., 1994; Lawrence et al., 2015). Assays were performed in T100 Thermal Cycler (Bio-Rad Laboratories Australia), initial denaturation at 95 °C for 30 s, followed by 35 cycles at 95 °C 15 s, 52 °C for 15 s, 68 °C for 1 min, then a final extension 68 °C for 5 min. Gel verified products of expected size (∼650 bp) were Sanger sequenced (Macrogen Inc., Seoul, Korea). Sequences were visually inspected and assembled using CLC Main Workbench 25.0.2 (CLC bio, Qiagen, Chadstone, Australia).

The gDNA obtained from ticks (n = 4) and fleas (n = 4) was submitted to Novogene (HK) Co., Ltd for indexing, library preparation and whole genome sequencing to generate approximately 5 Gb of sequencing data per sample. Sequencing was performed using Illumina sequencing platform with 150 bp paired-end sequencing chemistry. Raw FastQ files were processed in Galaxy Australia (https://usegalaxy.org.au/) (Galaxy Community, 2024)**.** Raw paired-end sequencing reads were examined for quality using *FastQC* (version 0.12.1) (Andrews, 2010) and trimmed using *Trimmomatic* (version 0.36.6) (Bolger et al., 2014) (parameters: “SLIDINGWINDOW:4:20”). The FastQ files were assembled into mitochondrial genomes using *GetOrganelle* (version 1.7.7.1) (Jin et al., 2020) (parameters: “-F animal_mt”) and annotated using *MITOS2* (version 2.1.10) (Donath et al., 2019) (parameters “--code 5 --refseqver refseq63m”). Sequences corresponding to mitochondrial DNA (mitogenome) were visually inspected and annotation manually adjusted using CLC Main Workbench (CLC bio, Qiagen, Chadstone, Australia).

For ticks, all of the *cox1* sequences labelled as *Ixodes tasmani* in NCBI GenBank were downloaded (Hammer et al., 2015; Ash et al., 2017; Kwak et al., 2017; Evans et al., 2019; Chandra and Šlapeta, 2020; Ghafar et al., 2023). In addition, the *cox1* sequences from the mitogenomes of other *Ixodes* species in NCBI GenBank were downloaded (Shao et al., 2004, 2005; Ash et al., 2017; Barker et al., 2022a, 2022b). Our *cox1* sequences were also compared against an unpublished database of mitogenomes to complement the morphological study of the few adult ticks (Barker, unpublished).

For fleas, *cox1* reference sequences were downloaded for available Australian endemic fleas: *Bradiopsylla echidnae, Stephanocircus pectinipes, S. dasyuri* and *Acanthopsylla jordani* (Kaewmongkol et al., 2011; Lawrence et al., 2014; Zhu et al., 2015). Sequences were aligned using CLC Main Workbench and FASTA alignments exported for phylogenetic analysis in MEGA12 (Kumar et al., 2024).

The final alignment of tick and flea *cox1* sequences consisted of 62 and 20 sequences, respectively. Phylogenetic trees and the best nucleotide substitution model was selected heuristically as one with the lowest Bayesian Information Criterion (BIC) in MEGA12 (Kumar et al., 2024). The evolutionary history of flea *cox1* was inferred using the Maximum Likelihood (ML) method with General Time Reversible model (GTR) of nucleotide substitutions with proportion of sites deemed evolutionarily invariant (+*I*). The percentage of replicate trees in which the associated taxa clustered together, where the number of replicates (n = 107) was determined adaptively. The flea *cox1* tree was rooted using *cox1* from a scorpionsfly, *Boreus borealis* Banks, 1923 (KU874461) (Sikes et al., 2017); molecular studies indicate that fleas (Siphonaptera) are a specialised, parasitic lineage of scorpionflies (Mecoptera) (Tihelka et al., 2020). For tick *cox1* alignment, the tree was reconstructed using the Minimum Evolution method (ME) with the evolutionary distances computed using the Maximum Composite Likelihood (MCL) method and the rate variation among sites was modelled with a gamma distribution (+*G*). Bootstrap test was based on 1000 replicates. The tick *cox1* tree was rooted using *cox1* from *Ixodes pavlovskyi* Pomerantzev, 1948 (NC_023831) mitogenome.

### 2.4 Molecular detection of *Rickettsia* and *Bartonella* from tick and flea samples

To screen tick and flea samples, a TaqMan probe-based real-time PCR (qPCR) targeting citrate synthase (∼75bp amplicon; *gltA*) gene to detect *Rickettsia* was used as previously adopted (Stenos et al., 2005; Šlapeta and Šlapeta, 2016) and a qPCR targeting transfer-messenger RNA (∼300bp amplicon; *ssrA*) was used to detect *Bartonella* as previously adopted (Diaz et al., 2012; Šlapeta and Šlapeta, 2016). *Rickettsia* primers and probe comprised forward primer: CS-F (S0576), 5′-TCG CAA ATG TTC ACG GTA CTT T-3′; reverse primer: CS-R (S0577) 5′-TCG TGC ATT TCT TTC CAT TGT G-3′ and probe CS-P (S0578), 5′-HEX-TGC AAT AGC AAG AAC CGT AGG CTG GAT G-3′-BHQ1. This assay will detect *Rickettsia* from the spotted fever and typhus group, but not the ancestral group, which contains *R. bellii* (Stenos et al., 2005). *Bartonella* primers and probe comprised forward primer: ssrA-F (S0508), 5′-GCT ATG GTA ATA AAT GGA CAA TGA AAT AA-3′; reverse primer: ssrA-R (S0509), 5′-GCT TCT GTT GCC AGG TG-3′ and probe S0510, 5′-FAM-ACC CCG CTT AAA CCT GCG ACG-3′-BHQ1. The reaction was carried out with 20 μl volume, and final concentration of primers was 400 nM and 100 nM of probes, with 4 μL of template DNA with Luna® Universal Probe qPCR Master Mix (New England Biolabs, Australia). Reactions were completed in duplicate on CFX96 Touch™ Real-Time PCR Detection System (BioRad, Australia), with initial denaturation 95 °C for 3 min, followed by 40 cycles of denaturation at 95 °C for 5 s, and annealing at 60 °C for 30 s. A negative and positive control was included.

A third real-time PCR assay was completed targeting the 16S rRNA gene, as a control to check the bacterial load in tick and flea samples, using the same protocol as described above. Generic bacterial primers and probe comprised forward primer: S0775, 5′-TCC TAC GGG AGG CAG CAG T-3′; reverse primer: S0776, 5′-GGA CTA CCA GGG TAT CTA ATC CTG TT-3′ and probe: S0777, 5′-FAM-BHQ1-CGT ATT ACC GCG GCT GCT GGC AC-3′ (Nadkarni et al., 2002).

Results for the *Rickettsia* and *Bartonella* runs were considered positive when duplicates yielded Ct values < 36, suspect positive when one or more repeats yielded Ct values ≥ 36 and negative if neither run crossed the threshold (Ct > 40) as previously adopted (Huang et al., 2021).

### 2.5 Data analysis

Fieldwork data sheets were transcribed into an Excel spreadsheet, where each row represented a single trap effort. Empty captures and by-catch were included for every site except Happy Jacks (as raw field sheets were damaged during wet weather and so only summary data was reliably obtained). Data was filtered to only include *B. parvus* on first capture and to remove any rows with blank values for age, weight and sex. To include by-catch trapping information, a series of columns were added for each species (*Rattus fuscipes, Mastacomys fuscus, Antechinus mimetes* and *A. agilis*). The ‘IF’ and ‘COUNTIFS’ formula was used so every row of data had the total number of by-catch captures reflected, specific to each site. To account for unequal trapping effort between sites, another set of columns was created to calculate the total number of captures per trap night, to give a comparable, site-specific value for each. A set of columns were created based on these values, using ’IF’ statements to assess each by-catch species presence or absence at sites.

RStudio software (version 2025.05.1 running R version 4.4.1) (Posit Team, 2025) was used to determine the *B. parvus* weight range, mean weight and standard deviation. Three generalised linear mixed models were created for tick presence, flea presence and tick load, using the ‘glmmTMB’ package (version 1.1.11) (McGillycuddy et al., 2025). The response variable of tick and flea presence/absence was modelled using a binomial error distribution, and tick load was modelled using negative binomial distribution (type ‘nbinom1’ in family function) to account for overdispersion. To investigate whether they influence parasite dynamics, *B. parvus* weight and sex were included in the model, as well as the remaining parasite variables, which were not the response variable in each model (tick and flea presence/absence and tick load). Age was not included as is likely related to weight and was difficult to accurately determine during field work. It was decided that weight was a more reliable, robust and objective measure. Inter-species interactions can influence parasite dynamics and so by-catch captures per trap night was used as an indicator of species biomass at each site, and *A. agilis* presence/absence was used as only one individual was captured. Sites are broadly categorised as either north or south sites due to known, broader differences (such as vegetation, boulderfield structure, climatic variables) and this variable was included to see if it influenced parasite dynamics. The mid-point elevation was used for each site and scaled so that a 1-unit increase was equal to 100m for meaningful interpretation. To account for distinct differences between sites, and any spatial clustering, site was included as a random effect in each model.

Model selection was conducted using backwards selection with the ‘drop1(full_model, test = "Chisq")’ function (package ‘stats’, version 4.4.1), on the full model with all fixed effect variables included, and site as a random effect. Backward elimination was used to obtain a parsimonious exploratory model, with awareness of its limitations and cautious interpretation of resulting inferences. Any variables with errors due to data insufficiency were removed, and the function was run again. Based on the output, fixed effect variables without significant values (p value < 0.05) were removed and the final model was assessed using the ‘summary()’ function. If any fixed effect variables were no longer significant, then ‘drop1()’ was run again. To assess improvement from the reduced models, the Akaike information criterion (AIC) values were used, and if minimal (AIC change <2), the simpler model was kept (Burnham and Anderson, 2002). If the ‘drop(1)’ function resulted in no fixed effect variables being significant, the variable closest to P-value of 0.05 was retained and the model run again, assessing AIC values as above, to select the best model. The ‘confint’ function (package ‘stats’, version 4.4.1) was used to assess the 95% confidence intervals for each of the three final models. Residual diagnostics were examined using the ‘DHARMa’ package and plots (Hartig, 2024), checking for any overdispersion, zero-inflation, non-uniformity and outliers and to ensure model assumptions were met.

### 2.6 Data accessibility

Tick and flea specimens were deposited to the Barker and Barker Collection at the University of Queensland under voucher IDs: B8795-B8796, B8971-B8989, B8991-B8994 and B9044-B9046. Data used for the statistical analysis and associated code as well as sequence alignment are available from LabArchives (https://dx.doi.org/10.25833/tz4v-gd74). The consensus sequence for each sample was submitted to GenBank (NCBI: National Centre for Biotechnology Information) under the accession numbers: PZ222055-PZ222087. Raw FastQ sequence data were deposited at SRA NCBI BioProject: PRJNA1437920 (https://www.ncbi.nlm.nih.gov/bioproject/PRJNA1437920).

## 3 Results

### 3.1 Baseline survey detects ticks and fleas on *Burramys parvus* in Kosciuszko National Park, New South Wales, Australia in 2024

There was a total of 3480 trap nights (one trap set overnight is equal to a single trap night): 1960 at southern Kosciuszko sites (400 at Charlotte Pass, 400 at Whites River, 400 at Summit Road, 300 at Paralyser and 460 at Blue Cow) and 1520 at northern Kosciuszko sites (400 at Snow Ridge Hill, 400 at Rough Creek and 720 at Happy Jacks). There was a total of 607 (387 female and 215 male) *B. parvus* captures. For each site, the total number of captures ranged between 21 and 206 (17-155 for female and 1-51 for males). A total of 257 (163 female, 91 male) unique *B. parvus* were captured, ranging between 11 and 108 (8-79 for females and 1-29 for males) for each site (Table 2).

**Table 2:** Tick and flea detection frequency, mean load (standard deviation) and samples taken from *Burramys parvus* in Kosciuszko National Park, Australia in October-November 2024.

Site	Tick									Flea					
	Detection frequency			Load mean (SD)			*B. parvus* sampled			Detection frequency			*B. parvus* sampled		
	All	F	M	All	F	M	All	F	M	All	F	M	All	F	M
All	120/257	73/163	47/91	3.6 (4.2)	4.0 (5.0)	3.1 (2.6)	47	20	27	32/257	14/163	18/91	15	4	11
	47%	45%	52%							12%	9%	20%			
Charlotte pass	14/25	8/13	6/12	1.9 (1.7)	1.3 (0.5)	2.8 (2.3)	8	3	5	2/25	0/13	2/12	0	0	0
	56%	62%	50%							8%	0%	17%			
Whites River	10/16	5/9	5/7	3.3 (3.7)	4.2 (5.0)	2.4 (2.1)	9	5	4	5/16	0/9	5/7	2	0	2
	63%	56%	71%							31%	0%	71%			
Summit Road	5/22	1/8	4/14	1.0 (0)	1.0 NA	1.0 (0)	6	1	5	1/22	0/8	1/14	0	0	0
	23%	13%	29%							5%	0%	7%			
Paralyser	8/27	3/18	5/9	3.8 (4.0)	1.7 (1.2)	5.0 (4.7)	4	0	4	6/27	3/18	3/9	3	1	2
	30%	17%	56%							22%	17%	33%			
Blue Cow	9/20	2/11	7/9	4.0 (5.1)	9.0 (11.3)	2.6 (1.5)	8	2	6	3/20	1/11	2/9	5	1	4
	45%	18%	78%							15%	9%	22%			
Snow Ridge Hill	61/108	45/79	16/29	4.3 (4.9)	4.5 (5.5)	3.8 (2.7)	6	5	1	14/108	9/79	5/29	4	1	3
	56%	57%	55%							13%	11%	17%			
Rough Creek	10/28	6/15	4/10	3.2 (2.3)	3.7 (2.7)	2.5 (1.7)	4	2	2	1/28	1/15	0/10	1	1	0
	36%	40%	40%							4%	7%	0%			
Happy Jacks	3/11	3/10	0/1	2.7 (1.5)	2.7 (1.5)	NA	2	2	0	0/11	0/10	0/1	0	0	0
	27%	30%	0%							0%	0%	0%			

**Note:** Detection frequency (number of *B. parvus* with parasites compared to unique individuals) and load only for first capture; number sampled is for any capture event; mean and standard deviation calculated for *B. parvus* with >0 ticks; Fleas either absent or present (no load). All = all *B. parvus* captured (both female and male), F = female, M = Male, SD = standard deviation, NA = not applicable.

A total of 142 and 38 *B. parvus* captures recorded observations of ticks and fleas respectively. Of *B*. *parvus* on their first capture, 47% and 12% were observed to have ticks and fleas, respectively (Table 2). Across all eight sites, *B. parvus* on their first capture had on average 3.6 (standard deviation, SD 4.2) ticks observed, with a total of 0-30 ticks each. In total there were 49 tick and 15 flea vials collected from 47 to 15 *B. parvus* individuals respectively (each vial represented all ticks or fleas collected from one capture event).

### 3.2 Presence of immature stages of ticks on *Burramys parvus*

There was a total of 113 tick specimens (two adults; 111 immatures stages - nymphs, larvae or damaged stages) from 47 *B. parvus* (Supplementary Table S1). Adult ticks were morphologically identified as *Ixodes* cf. *tasmani* (DOT250-1, voucher ID: B8796; DOT168-1, voucher ID: B8795) and were not processed for molecular identification; no *Ixodes heathi* were found.

**Fig. 2:**
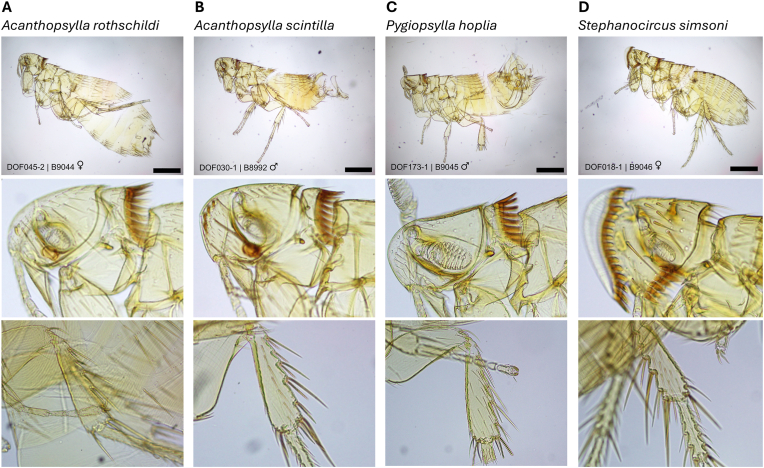
Representative images of the four fleas species identified during this study. Each flea was mounted and photographed, with three images of each species displayed: overall morphology at a scale of 500 μm, head and hind tibia with defining anatomy for species identification. A) Female *Acanthopsylla rothschildli* (DOF045-2; B9044); B) Male *Acanthopsylla scintilla* (DOF030-1; B8992); C) Male *Pygiopsylla hoplia* (DOF173-1; B9045); D) Female *Stephanocircus simsoni* (DOF018-1; B9046).

There was a total of 22 flea specimens from 15 *B. parvus* (Supplementary Table S1). Three genera were identified during initial morphological identification: *Acanthopsylla, Pygiopsylla* and *Stephanocircus.* From the subset of fleas (n = 19) that were cleared, four species were re-identified morphologically: *Acanthopsylla rothschildi* (Rainbow, 1905) (n = 5 female, 5 male, 1 unknown sex due to damaged condition), *Acanthopsylla scintilla* (Rothschild, 1936) (n = 1 male, DOF030-1), *Pygiopsylla hoplia* Jordan and Rothschild, 1922 (n = 2 male) and *Stephanocircus simsoni* Rothschild, 1905 (n = 5 female) (Fig. 2; Table 3).

**Table 3:** Molecular identification of tick and flea specimens and the host ID, site and associated morphological identification for each where relevant.

Site	Specimen ID^a^	Morphological ID	Molecular ID
Rough Creek	DOF018-1	*Stephanocircus simsoni* Rothschild, 1905	bNo match – new reference
Rough Creek	DOT024-1	-	*Ixodes* sp. cf. *tasmani* Neumann, 1899
	DOT024-2	-	*Ixodes* sp. cf. *tasmani* Neumann, 1899
	DOT024-3	-	*Ixodes* sp. cf. *tasmani* Neumann, 1899
Rough Creek	DOT025-1	-	b*Ixodes* sp. cf. *tasmani* Neumann, 1899
	DOT025-2	-	*Ixodes* sp. cf. *tasmani* Neumann, 1899
Snow Ridge Hill	DOF030-1	*Acanthopsylla scintilla* (Rothschild, 1936)	bNo match – new reference
	DOF030-2	*Stephanocircus simsoni* Rothschild, 1905	Off target amplification^c^
	DOF030-3	*Acanthopsylla rothschildi* (Rainbow, 1905)	No match – new reference
Snow Ridge Hill	DOT033-2	-	Off target amplification^c^
	DOT033-3	-	Ixodes sp. cf. tasmani Neumann, 1899
	DOT033-4	-	Ixodes sp. cf. tasmani Neumann, 1899
Snow Ridge Hill	DOF045-1	*Acanthopsylla rothschildi* (Rainbow, 1905)	No match – new reference
	DOF045-2	*Acanthopsylla rothschildi* (Rainbow, 1905)	bNo match – new reference
Snow Ridge Hill	DOF048-1	*Acanthopsylla rothschildi* (Rainbow, 1905)	Unsuccessful
Snow Ridge Hill	DOF063-1	*Acanthopsylla rothschildi* (Rainbow, 1905)	No match – new reference
Charlotte Pass	DOT155-2	-	Off target amplification^c^
Charlotte Pass	DOT165-1	-	*Ixodes* sp. cf. tasmani Neumann, 1899
Whites River	DOT173-1	-	*Ixodes* sp. cf. tasmani Neumann, 1899
	DOT173-2	-	*Ixodes* sp. cf. tasmani Neumann, 1899
	DOF173-1	*Pygiopsylla hoplia* Jordan and Rothschild, 1922	bNo match – new reference
Whites River	DOT174-2	-	*Ixodes* sp. cf. tasmani Neumann, 1899
Whites River	DOT184-1	-	*Ixodes* tasmani Neumann, 1899
Whites River	DOF185-1	*Acanthopsylla rothschildi* (Rainbow, 1905)	No match – new reference
Paralyser	DOF190-1	*Acanthopsylla rothschildi* (Rainbow, 1905)	No match – new reference
Paralyser	DOF199-1	*Pygiopsylla hoplia* Jordan and Rothschild, 1922	No match – new reference
Paralyser	DOT206-1		*Ixodes* sp. cf. tasmani Neumann, 1899
	DOF206-1	*Stephanocircus simsoni* Rothschild, 1905	Unsuccessful
Blue Cow	DOF218-1	*Acanthopsylla rothschildi* (Rainbow, 1905)	No match – new reference
	DOF218-2	*Acanthopsylla rothschildi* (Rainbow, 1905)	No match – new reference
Blue Cow	DOT228-1	-	Off target amplification^c^
Blue Cow	DOT229-2	-	*Ixodes* sp. cf. *tasmani* Neumann, 1899
	DOF229-1	*Acanthopsylla rothschildi* (Rainbow, 1905)	No match – new reference
Blue Cow	DOF230-1	*Stephanocircus simsoni* Rothschild, 1905	Unsuccessful
Blue Cow	DOT231-1	-	*Ixodes* sp. cf. *tasmani* Neumann, 1899
	DOF231-1	*Acanthopsylla rothschildi* (Rainbow, 1905)	No match – new reference
Blue Cow	DOT232-1	-	Off target amplification^c^
	DOT232-2	-	*Ixodes* sp. cf. *tasmani* Neumann, 1899
Blue Cow	DOF234-1	*Stephanocircus simsoni* Rothschild, 1905	Unsuccessful
Blue Cow	DOT235-1	-	bIxodes tasmani Neumann, 1899
Summit Road	DOT256-1	-	bIxodes sp. cf. tasmani Neumann, 1899
Summit Road	DOT257-1	-	bIxodes sp. cf. tasmani Neumann, 1899

a Specimen ID relates to host ID (ie. DOT257-1 the first tick [T] taken from possum 257, DOF045-2 is for the second flea [F] taken from possum number 45).

b Obtained via Illumina sequencing.

c successful amplification of *cox1* but off target host *Burramys parvus* match to DQ217582.

### 3.3 Molecular identification of *Ixodes tasmani* Neumann, 1899 and *Ixodes* sp. cf. *tasmani* Neumann, 1899 and three Australian flea species

Good quality total DNA was extracted from 27 ticks (one to four ticks from 17 *B. parvus* captures from across all eight collection sites; nymphs: n = 1, 1/18; larvae: n = 22, 22/83; damaged: n = 4, 4/10) and 19 fleas (one to three from the 15 *B. parvus* sampled from 5 sites), based on presence of amplified bacterial DNA (Ct < 35). There was successful amplification of ∼650bp of *cox1* gene from 85% (23/27) of tick and 74% (14/19) of flea DNA (Table 3). There were 19 (19/23) *cox1* sequences belonging to *Ixodes* ticks publicly available *cox1* sequences, >97% nucleotide identity; these ticks and respective *cox1* represent specimens from 14 *B. parvus* from seven out of the eight sampled localities (northern: Snow Ridge Hill and Rough creek; southern: Charlotte pass, Whites river, Summit road, Paralyser and Blue cow Kosciuszko) (Table 1, Table 3). The remaining four (4/27) *cox1* sequences matched host *cox1* sequence (DQ217582), indicating off target amplification of the marsupial host. For the majority (13/14) of the flea *cox1* sequences (that represent 10 *B. parvus* from four sites), there was no close match (>97% nucleotide identity) to available *cox1* sequences (Table 3). One *cox1* derived from flea DNA (1/14) matched the host (DQ217582).

**Fig. 3:**
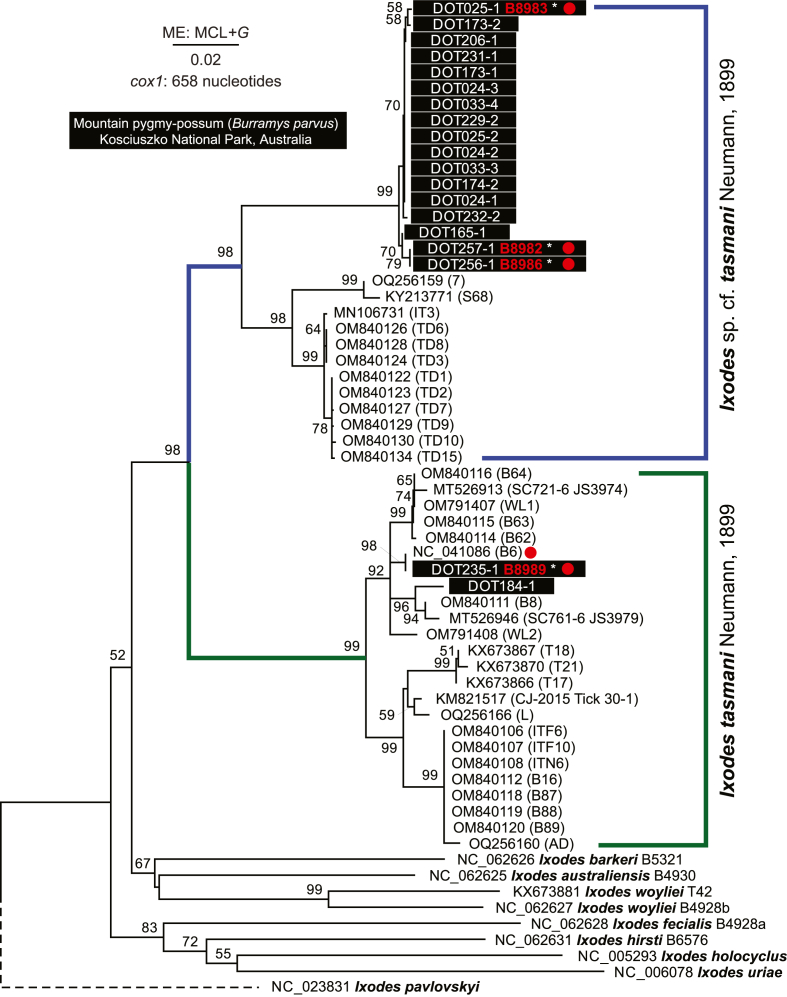
Phylogenetic analysis based on *cox1* nucleotide sequences from ticks, including *Ixodes tasmani* Neumann, 1899 and *Ixodes* sp. cf. *tasmani* obtained from *Burramys parvus* in Kosciuszko National Park, New South Wales, Australia. The tree was reconstructed using the Minimum Evolution (ME) method, and evolutionary distances were calculated using the Maximum Composite Likelihood (MCL) method. Rate variation among sites was modelled with a gamma distribution (+*G*). Bootstrap support values (1000 replicates) are shown at the corresponding nodes. The tree is drawn to scale, with branch lengths proportional to evolutionary distances. The alignment comprises 62 nucleotide sequences and 658 nucleotide (nt) positions. Accession numbers for publicly available sequences are provided alongside species names and voucher/isolate identifiers (in parentheses). Our sequences from ticks collected from *B. parvus* are highlighted with black rectangles and voucher ID from Barker and Barker collection in red (∗ indicates that short read whole genome sequence data are available, SRA NCBI BioProject: PRJNA1437920). Red circles indicate availability of mitogenome (mtDNA) that includes *cox1*. Two clades of *Ixodes tasmani* group ticks are colour-coded and labelled on the right. The mitogenome sequence NC_041086 (B6) is considered to be the *Ixodes tasmani* Neumann, 1899 (Barker & Cho, unpublished data). The sequence of *Ixodes pavlovskyi* (NC_023831) is included as the outgroup.

The tick *cox1* sequences were aligned with *Ixodes* spp. *cox1* sequences from GenBank, including *cox1* from a mitogenome of *Ixodes tasmani* (NC_041086). Phylogenetic analysis revealed consistent clustering of newly obtained *cox1* sequences with those of *I. tasmani* (Fig. 3). The *I. tasmani* cluster was strongly supported (98% bootstrap support) sister group cluster of *cox1* sequences derived from mitogenomes of *Ixodes barkeri* (NC_062626), *Ixodes australiensis* (NC_062625) and *Ixodes woyliei* (NC_062627); however, the monophyly of these two clusters was weak (52% bootstrap support). The *I. tasmani* cluster was split into two sister groups representing (i) *I. tasmani* and (ii) *Ixodes* sp. cf*. tasmani*; the newly obtained *cox1* sequences from this study clustered within both groups (Fig. 3). Most of the *cox1* sequences from this study formed an independent (>95% bootstrap support) group within the *Ixodes* sp. cf*. tasmani* group (Fig. 3). For one representative of *Ixodes tasmani* group (voucher ID: B8989) and three representatives of *Ixodes* sp. cf*. tasmani* group (voucher ID: B8982, B8983, B8986) short read (150-bp pair end) whole genome sequence data was generated and complete mitogenomes were assembled. Nucleotide percent identity between the mitogenome of *I. tasmani* (voucher ID: B8989) and *Ixodes* sp. cf*. tasmani* group (voucher ID: B8982, B8983, B8986) was 85%, while *I. tasmani* (voucher ID: B8989) compared to the reference mitogenome *I. tasmani* (NC_041086) was 98%.

**Fig. 4:**
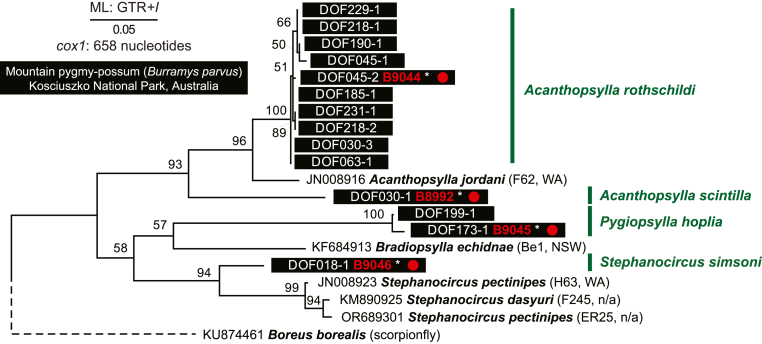
Phylogenetic analysis based on *cox1* nucleotide sequences from fleas (Siphonaptera) collected from *Burramys parvus* in Kosciuszko National Park, New South Wales, Australia. The tree was reconstructed using the Maximum Likelihood method and the General Time Reversible (GTR) model of nucleotide substitution with a proportion of evolutionarily invariant sites (+*I*). Bootstrap support, obtained adaptively (104 replicates), is shown at the corresponding nodes. The tree is drawn to scale, with branch lengths proportional to evolutionary distances. The alignment comprises 20 nucleotide sequences and 658 nucleotide (nt) positions. Accession numbers for publicly available sequences are provided together with species names and voucher/isolate identifiers and locations, where available (not available = n/a). Newly generated sequences from fleas collected from *B. parvus* are highlighted with black rectangles and voucher ID from Barker and Barker collection in red (∗ indicates that short read whole genome sequence data are available, SRA NCBI BioProject: PRJNA1437920). Red circles indicate availability of mitogenome (mtDNA) that includes *cox1*. Several publicly available *cox1* sequences assigned to *Stephanocircus* spp. are likely misidentified, and their taxonomic status should be re-examined. The sequence of the scorpionfly *Boreus borealis* (KU874461) is used as the outgroup.

Ten *Acanthopsylla rothschildi*, two *Pygiopsylla hoplia* and one *Acanthopsylla scintilla cox1* was obtained using a Sanger sequencing approach. Initially, none of the *Stephanocircus simsoni* samples yielded *cox1* PCR product for Sanger sequencing. For one representative of each flea species (voucher ID: B8992, B9044, B9045), including *S. simsoni* (voucher ID: B9046), short read (150-bp pair end) whole genome sequence data was generated and complete or almost complete mitogenomes were assembled. All new *cox1* sequences from this study were aligned with available Australian endemic flea species. The phylogenetic tree revealed monophyly of *Acanthopsylla* spp. sequences (93%) and *Stephanocircus* spp. sequences (94%) (Fig. 4).

### 3.4 Absence of *Bartonella* and low-level detection of *Rickettsia* using real-time PCR

The real-time PCR targeting *Bartonella* was negative for all tick and flea samples examined (19/19 and 27/27 respectively). The real-time PCR targeting *Rickettsia* was positive for one flea DNA sample (voucher ID: B9045, DOF173-1; Ct < 36), and one flea DNA sample (voucher ID: B9046, DOF018-1) was considered suspect for *Rickettsia,* based on a Ct value > 36 (Supplementary Table S1). All non-target controls were negative, positive controls were positive, and all samples were positive during the generic bacteria assay.

### *3.5* *Burramys parvus* body weight inversely related to tick presence and load, and higher odds of flea presence for male hosts

To identify host and habitat factors associated with parasite dynamics, statistical analysis was completed to examine whether variation in tick presence, flea presence, and tick load could be explained by differences in host traits or environmental characteristics. The *B*. *parvus* individuals captured in this study weighed 29–58 g (mean = 40 g; SD = 6 g). The final models showed that only host factors, body weight and sex, were associated with ectoparasite patterns, whereas the tested environmental and by-catch variables did not improve model fit.

To determine whether tick occurrence was associated with host or site characteristics, tick presence was modelled as a function of weight, sex, flea presence, tick load, by-catch activity, *A. agilis* presence, elevation, and north/south site category. After model selection, weight was the only retained predictor. Heavier individuals had lower odds of carrying ticks: each 1 g increase in body weight (≈2.5% of mean mass) was associated with a 5.8% reduction in the odds of tick presence (OR = 0.94, 95% CI: 0.90–0.99, p = 0.017). Site-level variation accounted for a random-effect variance of 0.10 (SD = 0.30). This indicates that tick presence appears to be more strongly associated with host condition (weight) than with habitat differences measured across sites.

To test whether flea occurrence was associated with the same host or site variables, flea presence was modelled using the same full set of predictors. Model selection retained sex as the single fixed effect and males had 2.6 times higher odds of flea presence than females (OR = 2.62, 95% CI: 1.19-5.77, p = 0.017). The random effect of site explained a variance of 0.20 (SD = 0.50). Thus, flea presence differed between sexes but showed no detectable association with habitat or co-infection variables.

Finally, the number of ticks on an individual was modelled with the same host and habitat predictors. As with tick presence, weight was the only retained fixed effect. Each 1 g increase in weight corresponded to a 3.9% reduction in tick load (OR = 0.96, 95% CI: 0.93-1.00, p = 0.031). Site explained a variance of 0.05 (SD = 0.20). This suggests that tick burden, like tick presence, appears more strongly associated with host weight than environmental factors. Diagnostics indicated that assumptions were met for all three models, with no evidence of zero inflation, overdispersion, or non-uniform residuals.

## 4 Discussion

Parasitofauna of the critically endangered mountain pygmy-possum (*B. parvus*) has not been systematically evaluated, despite intensive monitoring across its highly restricted distribution range, where Archer et al. (2019) outlines the conservation plan for this threatened species. To begin to fill this knowledge gap, we applied a rigorous sampling strategy to collect data, ticks and fleas alongside the annual monitoring survey of *B*. *parvus* in Kosciuszko National Park, NSW. Molecular identification aligned with morphological identification, and new reference sequences, including short-read Illumina whole genome sequence data, for two tick taxa and four flea species were obtained. The *B. parvus* with lower weight had higher odds of tick presence and load, and male *B. parvus* had higher odds of flea presence. We consider a number of factors that could be behind these statistical findings, whilst acknowledging further research is required to ascertain the drivers behind these associations.

Two flea species have been reported previously on *B. parvus,* specifically *Stephanocircus simsoni* and *Acanthopsylla rothschildi rothschildi* (Dunnet and Mardon, 1974). This helmet flea (*S*. *simsoni*) is a generalist found on native Australian wildlife (small mammals) in the southern states of Victoria and Tasmania, but has also been found in the Australian Capital Territory, north of our study sites (Dunnet and Mardon, 1974). The only record of the helmet-flea *S. simsoni* on *B. parvus* was from Victoria (Dunnet and Mardon, 1974), making those identified in this study the first to be reported from the restricted NSW localities of *B. parvus*. Existing records note that *A. r. rothschildi* has been found on *B. parvus* from Kosciuszko National Park, NSW (Dunnet and Mardon, 1974) and our study has identified a further 11 *A. rothschildi* fleas from *B. parvus* in Kosciuszko*.* We further identified *A. scintilla* (DOF030-1) for which there are currently three subspecies: *A. scintilla rectangulata, A. scintilla scintilla* or *A. scintilla tasmanica* (Dunnet and Mardon, 1974)*.* There were two samples identified as *Pygiopsylla hoplia* (DOF173-1, DOF199-1) from this study, the first reports of this flea on this host species. The helmet flea (*S. simsoni*) and fleas within the *Acanthopsylla* and *Pygiopsylla* genera have a wide host range, including species sharing *B. parvus* habitat. This study contributes the first complete and almost complete mitogenomes for these four flea species and provides new reference sequences for *cox1* from the fleas that underwent molecular analysis during this study.

Other mammal species recorded in localities shared with *B. parvus* include *Rattus fuscipes*, *Mastacomys fuscus*, *Antechinus mimetes*, and *A. agilis*. They are all known to harbour a range of flea species (Dunnet and Mardon, 1974). Because these are common by-catch within *B. parvus* habitats, it is likely *B. parvus* encounter their nests, facilitating the exchange and development of fleas among co-occurring hosts. The flea *A. rothschildi* has been reported on the agile antechinus (*A. agilis*), a small carnivorous marsupial native to Australia (Weaver and Aberton, 2004). The helmet fleas *Stephanocircus dasyuri* and *S. pectinipes* have been reported on the broad-toothed rat (*M. fuscus*) (Dunnet and Mardon, 1974), while *Pygiopsylla hoplia* has been recorded from a range of carnivorous marsupials and native rodents, including *M. fuscus*, *A. agilis*, and *R. fuscipes* (Hastriter, 2021). The bush rat (*R. fuscipes*) has been reported to carry *Stephanocircus pectinipes*, *Acanthopsylla scintilla rectangulata*, *A. pavida*, *S. concinnus*, *S. dasyuri*, *Pygiopsylla gravis*, *P. iridis*, *P. rainbowi*, and *P. sinuate* (Dunnet and Mardon, 1974). Records for the dusky antechinus (*A. mimetes*) include *S. greeni tasmanica*, *P. rainbowi*, *A. dunneti*, *S. dasyuri*, and *S. greeni greeni* (Dunnet and Mardon, 1974; Hastriter, 2021). The diversity of flea species documented on native mammals sharing habitat with *B. parvus* highlights the range of ectoparasites that may contribute to the transmission of vector-borne infectious agents in these ecosystems.

*Rickettsia* and *Bartonella* are notorious vector borne bacteria potentially affecting host welfare (Moore et al., 2026). Current results suggest a negligible presence of *Rickettsia* and *Bartonella* in ectoparasites collected from *B. parvus* in Kosciuszko National Park*.* There was no *Bartonella* found in any of the samples screened and only two flea sample were potentially positive for *Rickettsia* DNA. Based on the number of samples analysed, there is 90% confidence that the prevalence of either agent does not exceed greater than five percent across the populations studied. Further PCR assays targeting more variable loci, followed by sequencing and alignment against reference sequences within GenBank (as previously described) (Hii et al., 2015), would be required to determine the species of *Rickettsia*. The assay used to screen for *Rickettsia* is highly specific and is not expected to amplify *Rickettsia bellii*, a strain that predates the divergence of the spotted fever and typhus groups (Stothard et al., 1994) and is found in the United States and parts of South America (Parola et al., 2013). Future monitoring of *B. parvus* should be expanded to screen for common vector-borne infectious agents, whilst also screening other hosts and sampling during different seasons to help researchers understand if the low levels found during this study are comparable across different species or climatic conditions.

The marsupial tick *Ixodes tasmani* Neumann, 1899 is a widely distributed generalist species that parasitises a range of Australian marsupials (Barker and Barker, 2023). After engorgement, adult females drop from their hosts and deposit hundreds of eggs, which hatch into highly mobile larvae that actively seek small mammal hosts (Barker and Barker, 2023). The high prevalence of larvae and nymphs on approximately 50% of *B. parvus* individuals suggests that this species represents a highly suitable host for immature stages, either as a primary or secondary host. In contrast, adult *I. tasmani* most likely prefer larger marsupials and perhaps these larger hosts are contaminating *B. parvus* habitat with engorged adult females that subsequently lay eggs. The survey team frequently notes large, engorged ticks are present on bush rats (*R. fuscipes*) and dusky antechnius (*A. mimetes*)*.* Unlike flea infestations, which are concentrated in dens, tick encounters for *B. parvus* are likely to occur in foraging areas (Hart and Hart, 2018).

Interestingly, the ticks collected in this study appear to belong to two taxa groups that can be recognised as *I. tasmani* Neumann, 1899, and a closely related but molecularly distinct lineage currently referred to as *Ixodes* sp. cf. *tasmani*. This molecular heterogeneity within what has traditionally been recognised as *I. tasmani* has been documented in several studies over the past decade examining tick diversity in Australia (Shao et al., 2004, 2005; Hammer et al., 2015; Ash et al., 2017; Kwak et al., 2017; Evans et al., 2019; Chandra and Šlapeta, 2020; Barker et al., 2022a, 2022b; Ghafar et al., 2023). However, the biological or ecological significance beyond genetic divergence remains uncertain and requires further investigation.

A specialist tick, *Ixodes heathi*, was recently described from immature stages collected from *B. parvus* on the Victorian side of the host species’ alpine distribution (Kwak et al., 2018). The absence of *I. heathi* in our comprehensive survey across Kosciuszko National Park sites suggests that this tick may be absent from NSW *B. parvus* populations, or alternatively, that the adult-stage host does not occur in, or does not regularly visit, these NSW habitats and therefore does not introduce the tick into these areas. If the latter explanation is correct, then *I. heathi* may not be a *B. parvus* specialist as initially suggested (Kwak et al., 2018).

From this study, it is unclear if decreased body weight is the result, or cause, of increased tick presence, or, if it is due to another factor. *Burramys parvus* may have decreased body condition due to direct effects of parasitism, as a previous study conducted on tammar wallabies (*Macropus eugenii*), found the total gastrointestinal parasite load was strongly linked to body condition (Robert and Schwanz, 2013). However, a large meta-analysis warned against the frequent use of parasite burdens as a measure of host health or body condition, as relationships can vary greatly in strength and direction, and there is often bias towards associating increased parasite loads with poor body condition (Sánchez et al., 2018). It may be that smaller *B. parvus* are facing other pressures, or are in poor health and therefore have poor grooming behaviour, which has led to increased parasite burdens, as has been previously documented in other mammals (Akinyi et al., 2013; Hart and Hart, 2018). On the other hand, there may be a presently unknown factor that is shared between *B*. *parvus* of lower weight and those with ticks, that has not been accounted for in this dataset. The exclusion of age and host reproductive status from the modelling in this study may bias interpretation and future studies should ensure this variable is recorded consistently to allow inclusion in statistical analysis. A study, conducted on lizards from the same region sampled during this present study, found ectoparasite abundance decreased with increased elevation, suggesting climate change may lead to altered parasite dynamics (Hamilton et al., 2021). However, elevation and north versus south site variables were not found to be significant influencers of parasite presence or load in models created during this study. Geiser and Broome (1991) found that not all juvenile *B. parvus* hibernate in captivity during winter and Broome (2001) observed lighter weight individuals remained active for longer at lower, vegetated elevations on Mt Blue Cow, suggesting that light weight individuals may not hibernate in the wild. This could explain the higher tick loads on light weight individuals in November-December and yet further research would be required beyond the findings of this study, to ascertain the direct causes behind these statistical findings.

The statistical finding that male *B. parvus* had higher odds of flea presence may suggest behavioural or immunological differences between the sexes. Male *B. parvus* disperse to lower elevation sites and tend to inhabit areas with higher vegetation coverage where bush rats are also most abundant (Broome, 2001; Broome et al., 2013), which may be a driver behind this finding. Some suggest male-biased parasitism may be due to suppressive effects of androgens (male sex hormones) on immune system function (Kowalski et al., 2015). For example, a study assessing flea differences between sexes of three rodent species (*Apodemus agrarius, Apodemus flavicollis,* and *Myodes glareolus*) concluded that both spatial and immunological drivers were behind the differences seen in these species (Kowalski et al., 2015). There may also be sex-based differences in inter-species interactions. Although by-catch information was included in statistical analysis, these variables were not statistically significant, perhaps due to the simplicity of the variables (using capture number as a proxy for biomass). In future surveys, collecting data on by-catch species sex, weight, number of unique individuals and any parasites present would provide deeper insight into whether inter-species interactions influence parasite dynamics of *B. parvus.* As fleas tend to be concentrated in burrows or animal nests, the increased odds of flea presence on males may be indicative of different nest conditions or behavioural use of nests for male *B. parvus*. Broome (2001) found that radio tracked male *B. parvus* frequently shared nest sites either concurrently, or on separate nights during November-December, when females have individual natal nests. Further research is required, ideally over a longer period, to understand why males were more strongly associated with flea presence in this study. It would also be instructive to conduct parasitological studies at other times of year to ascertain if parasite dynamics change with seasonal changes in host behaviour and to investigate the effects of warming temperatures on hibernation propensity, parasite loads and community composition.

## 5 Conclusions

This study provides a comprehensive assessment of ticks and fleas and associated *Rickettsia* and *Bartonella* in *B. parvus* across key sites in Kosciuszko National Park during October-November, offering a strong baseline for interpreting parasite dynamics in this critically endangered marsupial. Host characteristics, particularly body weight and sex, were the main factors associated with variation in parasite presence and load, whereas the environmental variables examined did not meaningfully explain these patterns within the study area. Several ectoparasite species not previously documented from *B. parvus* were identified, and *Bartonella* and *Rickettsia* were detected at low prevalence, highlighting the value of continued health surveillance.

As translocations, climate-driven shifts, and long-term monitoring remain central to *B. parvus* conservation, repeated parasite surveys will be important for detecting temporal changes in host-parasite relationships. Integrating parasite and pathogen monitoring into ongoing management programs will improve understanding of host health, behaviour, and ecological pressures, helping guide evidence-based strategies for the conservation of this critically endangered species.

## CRediT authorship contribution statement

**Danielle J. Oste:** Writing – review & editing, Writing – original draft, Visualization, Project administration, Methodology, Investigation, Formal analysis, Data curation, Conceptualization. **Damien P. Higgins:** Writing – review & editing, Validation, Supervision, Resources, Project administration, Methodology, Funding acquisition, Conceptualization. **Hayley Bates:** Writing – review & editing, Supervision, Methodology, Investigation, Conceptualization. **Linda Broome:** Writing – review & editing, Resources, Methodology, Investigation. **Floris F. van Ogtrop:** Writing – review & editing, Methodology, Investigation, Formal analysis, Data curation. **Mingeun Cho:** Writing – review & editing, Formal analysis. **Stephen C. Barker:** Writing – review & editing, Resources, Investigation, Formal analysis. **Jan Šlapeta:** Writing – review & editing, Validation, Supervision, Resources, Methodology, Investigation, Formal analysis, Conceptualization.

## Funding

This work was supported by the 10.13039/501100001774University of Sydney through research funding gained as part of the Honours program.

## Declaration of competing interest

The authors declare the following financial interests/personal relationships which may be considered as potential competing interests: Advisory Group of the International Journal for Parasitology: Parasites and Wildlife (JŠ). Other authors declare that they have no known competing financial interests or personal relationships that could have appeared to influence the work reported in this paper.
