# Supplementary material for: Ticks and fleas of the critically endangered mountain pygmy-possum (Burramys parvus) in Kosciuszko National Park, Australia
Source: Int J Parasitol Parasites Wildl. 2026 Apr 17;30:101232. doi: 10.1016/j.ijppaw.2026.101232 (PMC13125916; doi:10.1016/j.ijppaw.2026.101232)
Supplement: Multimedia component 1 [file mmc1.docx]

### Supplementary material:

**Supplementary Table S1:** Summary of tick and flea specimens collected from *Burramys parvus*, including capture information, parasite observations, sample information, morphological and molecular identification and the result of screening for *Rickettsia* and *Bartonella* where relevant.

| ***Burramys parvus* capture information** | | | | | | | | | **Parasite Observations** | | | | | **Sample Information** | | | | | | | | | |
| --- | --- | --- | --- | --- | --- | --- | --- | --- | --- | --- | --- | --- | --- | --- | --- | --- | --- | --- | --- | --- | --- | --- | --- |
| ***Burramys parvus* ID** | **Sex** | **Weight (g)** | **Age** | **Testes Size (cm)** | **Female with young** | **Number of young** | **Length of Young (cm)** | **Site** | **Capture Event** | **Date** | **Trap** | **Observed ticks** | **Observed fleas** | **Specimen ID** | **Barker collection ID** | **Tick Or Flea** | **Tick Stage** | **Flea Sex** | **Morphological ID** | **Molecular ID** | ***Rickettsia*** | ***Bartonella*** |  |
| Bp_001 | F | 31 | A | - | Y | 3 | 0.8 | RC | 2 | 23/10/24 | RCEM-12 | 1 | N | DOT001-1 |  | T | N | - | - | - | - | - |  |
| Bp_017 | M | 36 | A | 1.1 | - | - | - | RC | 1 | 23/10/24 | RCE-19 | 2 | N | DOT017-1 |  | T | L | - | - | - | - | - |  |
| Bp_018 | F | 30 | A | - | Y | 4 | 0.3 | RC | 1 | 23/10/24 | RCE-28 | 3 | Y | DOF018-1 | B9046 | F | - | F | S.s. | Uns. | S | N |  |
| Bp_024 | F | 31 | A | - | Y | 4 | 0.3 | RC | 1 | 24/10/24 | RCE-21 | 7 | N | DOT024-1 | B8985 | T | L | - | - | I. sp. cf. t. | N | N |  |
|  |  |  |  |  |  |  |  |  |  |  |  |  |  | DOT024-2 | B8974 | T | L | - | - | I. sp. cf. t. | N | N |  |
|  |  |  |  |  |  |  |  |  |  |  |  |  |  | DOT024-3 | B8979 | T | L | - | - | I. sp. cf. t. | N | N |  |
|  |  |  |  |  |  |  |  |  |  |  |  |  |  | DOT024-4 |  | T | L | - | - | - | - | - |  |
| Bp_025 | M | 40 | A | 1.2 | - | - | - | RC | 1 | 24/10/24 | RCE-28 | 5 | N | DOT025-1 | B8983 | T | L | - | - | I. sp. cf. t. | N | N |  |
|  |  |  |  |  |  |  |  |  |  |  |  |  |  | DOT025-2 | B8975 | T | L | - | - | I. sp. cf. t. | N | N |  |
| Bp_030 | M | 41 | A | 1 | - | - | - | SRH | 1 | 29/10/24 | A-6 | 5 | Y | DOF030-1 | B8992 | F | - | M | A. s. | NM | N | N |  |
|  |  |  |  |  |  |  |  |  |  |  |  |  |  | DOF030-2 |  | F | - | F | S.s. | B.p. | N | N |  |
|  |  |  |  |  |  |  |  |  |  |  |  |  |  | DOF030-3 |  | F | - | M | A. r. | NM | N | N |  |
| Bp_032 | F | 33 | A | - | D4 |  |  | SRH | 1 | 29/10/24 | A-8 | 5 | N | DOT032-1 |  | T | N | - | - | - | - | - |  |
|  |  |  |  |  |  |  |  |  |  |  |  |  |  | DOT032-2 |  | T | L | - | - | - | - | - |  |
|  |  |  |  |  |  |  |  |  |  |  |  |  |  | DOT032-3 |  | T | L | - | - | - | - | - |  |
| Bp_033 | F | 34 | S | - | Y | 4 | 1 | SRH | 1 | 29/10/24 | A-12 | 9 | N | DOT033-1 |  | T | L | - | - | Uns. | N | N |  |
|  |  |  |  |  |  |  |  |  |  |  |  |  |  | DOT033-2 |  | T | L | - | - | B.p. | N | N |  |
|  |  |  |  |  |  |  |  |  |  |  |  |  |  | DOT033-3 | B8973 | T | L | - | - | I. sp. cf. t. | N | N |  |
|  |  |  |  |  |  |  |  |  |  |  |  |  |  | DOT033-4 | B8978 | T | L | - | - | I. sp. cf. t. | N | N |  |
|  |  |  |  |  |  |  |  |  |  |  |  |  |  | DOT033-5 |  | T | L | - | - | - | - | - |  |
|  |  |  |  |  |  |  |  |  |  |  |  |  |  | DOT033-6 |  | T | L | - | - | - | - | - |  |
|  |  |  |  |  |  |  |  |  |  |  |  |  |  | DOT033-7 |  | T | L | - | - | - | - | - |  |
|  |  |  |  |  |  |  |  |  |  |  |  |  |  | DOT033-8 |  | T | L | - | - | - | - | - |  |
| Bp_034 | F | 42 | A | - | Y | 4 | 1.5 | SRH | 1 | 29/10/24 | A-19 | 5 | N | DOT034-1 |  | T | L | - | - | - | - | - |  |
|  |  |  |  |  |  |  |  |  |  |  |  |  |  | DOT034-2 |  | T | L | - | - | - | - | - |  |
|  |  |  |  |  |  |  |  |  |  |  |  |  |  | DOT034-3 |  | T | L | - | - | - | - | - |  |
|  |  |  |  |  |  |  |  |  |  |  |  |  |  | DOT034-4 |  | T | L | - | - | - | - | - |  |
| Bp_035 | F | 39 | A | - | Y | 4 | 1.3 | SRH | 1 | 29/10/24 | A-22 | 1 | N | DOT035-1 |  | T | N | - | - | - | - | - |  |
| Bp_038 | F | 45 | A | - | Y | 4 | 1.5 | SRH | 1 | 29/10/24 | A-26 | 5 | N | DOT038-1 |  | T | N | - | - | - | - | - |  |
| Bp_045 | M | 39 | A | 1.0 | - | - | - | SRH | 1 | 29/10/24 | A-37 | 0 | Y | DOF045-1 |  | F | - | M | A. r. | NM | N | N |  |
|  |  |  |  |  |  |  |  |  |  |  |  |  |  | DOF045-2 | B9044 | F | - | F | A. r. | NM | N | N |  |
|  |  |  |  |  |  |  |  |  |  |  |  |  |  | DOF045-3 |  | F | - | - | A. | - | - | - |  |
|  |  |  |  |  |  |  |  |  |  |  |  |  |  | DOF045-4 |  | F | - | - | A. | - | - | - |  |
|  |  |  |  |  |  |  |  |  |  |  |  |  |  | DOF045-5 |  | F | - | - | A. | - | - | - |  |
| Bp_048 | M | 36 | A | 1.0 | - | - | - | SRH | 1 | 29/10/24 | A-41 | 3 | Y | DOF048-1 |  | F | - | F | A. r. | Uns. | N | N |  |
| Bp_063 | F | 39 | A | - | Y | 4 | 0.6 | SRH | 2 | 30/10/24 | B-33 | 0 | Y | DOF063-1 |  | F | - | U | A. r. | NM | N | N |  |
| Bp_080 | M | 35 | A | 0.8 | - | - | - | SRH | 2 | 31/10/24 | A-32 | 7 | N | DOT080-1 |  | T | N | - | - | - | - | - |  |
|  |  |  |  |  |  |  |  |  |  |  |  |  |  | DOT080-2 |  | T | N | - | - | - | - | - |  |
| Bp_137 | F | 46 | A | - | NC |  |  | HJ | 1 | 16/11/24 | HJ35Trap1:16 | 1 | N | DOT137-1 |  | T | D | - | - | Uns. | N | N |  |
| Bp_139 | F | 48 | A | - | Y | >2 | 1 | HJ | 1 | 16/11/24 | HJ35Trap1:30 | 4 | N | DOT139-1 |  | T | L | - | - | - | - | - |  |
|  |  |  |  |  |  |  |  |  |  |  |  |  |  | DOT139-2 |  | T | D | - | - | - | - | - |  |
| Bp_155 | F | 41 | A |  | Y | 4 | 0.8 | CP | 1 | 16/11/24 | C-10 | 2 | N | DOT155-1 |  | T | L | - | - | - | - | - |  |
|  |  |  |  |  |  |  |  |  | 3 | 18/11/24 | C-10 | 1 | N | DOT155-2 |  | T | L | - | - | B.p. | N | N |  |
| Bp_158 | F | 41 | A | - | OG |  |  | CP | 1 | 16/11/24 | C-23 | 1 | N | DOT158-1 |  | T | L | - | - | - | - | - |  |
| Bp_162 | M | 49 | S | 0.8 | - | - | - | CP | 1 | 16/11/24 | D-10 | 2 | N | DOT162-1 |  | T | L | - | - | - | - | - |  |
| Bp_163 | M | 47 | S | 1.0 | - | - | - | CP | 1 | 16/11/24 | D-13 | 4 | N | DOT163-1 |  | T | D | - | - | - | - | - |  |
|  |  |  |  |  |  |  |  |  |  |  |  |  |  | DOT163-2 |  | T | L | - | - | - | - | - |  |
|  |  |  |  |  |  |  |  |  |  |  |  |  |  | DOT163-3 |  | T | L | - | - | - | - | - |  |
|  |  |  |  |  |  |  |  |  |  |  |  |  |  | DOT163-4 |  | T | L | - | - | - | - | - |  |
|  |  |  |  |  |  |  |  |  |  |  |  |  |  | DOT163-5 |  | T | L | - | - | - | - | - |  |
| Bp_164 | M | 45 | A | 1.0 | - | - | - | CP | 1 | 16/11/24 | D-14 | 2 | N | DOT164-1 |  | T | L | - | - | - | - | - |  |
|  |  |  |  |  |  |  |  |  |  |  |  |  |  | DOT164-2 |  | T | L | - | - | - | - | - |  |
|  |  |  |  |  |  |  |  |  |  |  |  |  |  | DOT164-3 |  | T | L | - | - | - | - | - |  |
| Bp_165 | M | 54 | A | 1.0 | - | - | - | CP | 1 | 16/11/24 | D-17 | 1 | N | DOT165-1 | B8987 | T | D | - | - | I. sp. cf. t. | N | N |  |
| Bp_167 | M | 40 | S | 0.4 | - | - | - | CP | 1 | 16/11/24 | D-23 | 7 | N | DOT167-1 |  | T | L | - | - | - | - | - |  |
|  |  |  |  |  |  |  |  |  |  |  |  |  |  | DOT167-2 |  | T | L | - | - | - | - | - |  |
|  |  |  |  |  |  |  |  |  |  |  |  |  |  | DOT167-3 |  | T | L | - | - | - | - | - |  |
|  |  |  |  |  |  |  |  |  |  |  |  |  |  | DOT167-4 |  | T | L | - | - | - | - | - |  |
|  |  |  |  |  |  |  |  |  |  |  |  |  |  | DOT167-5 |  | T | N | - | - | - | - | - |  |
|  |  |  |  |  |  |  |  |  |  |  |  |  |  | DOT167-6 |  | T | N | - | - | - | - | - |  |
| Bp_168 | F | 43 | A | - | Y | 4 | 0.5 | CP | 2 | 18/11/24 | A-24 | 1 | N | DOT168-1 | B8795 | T | A | - | I. cf. t. | - | - | - |  |
| Bp_173 | M | 37 | A | 0.5 | - | - | - | WR | 1 | 16/11/24 | B-5 | 6 | Y | DOT173-1 | B8980 | T | L | - | - | I. sp. cf. t. | N | N |  |
|  |  |  |  |  |  |  |  |  |  |  |  |  |  | DOT173-2 | B8972 | T | L | - | - | I. sp. cf. t. | N | N |  |
|  |  |  |  |  |  |  |  |  |  |  |  |  |  | DOT173-3 |  | T | L | - | - | - | - | - |  |
|  |  |  |  |  |  |  |  |  |  |  |  |  |  | DOF173-1 | B9045 | F | - | M | P. h. | NM | P | N |  |
| Bp_174 | F | 38 | A | - | Y | 4 | 0.7 | WR | 1 | 16/11/24 | B-6 | 13 | N | DOT174-1 |  | T | L | - | - | Uns. | N | N |  |
|  |  |  |  |  |  |  |  |  |  |  |  |  |  | DOT174-2 | B8971 | T | L | - | - | I. sp. cf. t. | N | N |  |
|  |  |  |  |  |  |  |  |  |  |  |  |  |  | DOT174-3 |  | T | L | - | - | - | - | - |  |
|  |  |  |  |  |  |  |  |  |  |  |  |  |  | DOT174-4 |  | T | L | - | - | - | - | - |  |
| Bp_177 | F | 42 | A | - | Y | 4 | 1 | WR | 1 | 16/11/24 | B-19 | 1 | N | DOT177-1 |  | T | N | - | - | - | - | - |  |
| Bp_179 | F | 55 | A | - | Y | 4 | 1.2 | WR | 1 | 17/11/24 | B-6 | 3 | N | DOT179-1 |  | T | L | - | - | - | - | - |  |
|  |  |  |  |  |  |  |  |  |  |  |  |  |  | DOT179-2 |  | T | L | - | - | - | - | - |  |
|  |  |  |  |  |  |  |  |  |  |  |  |  |  | DOT179-3 |  | T | L | - | - | - | - | - |  |
| Bp_180 | M | 33 | A | 0.4 | - | - | - | WR | 1 | 17/11/24 | B-14 | 2 | N | DOT180-1 |  | T | L | - | - | - | - | - |  |
|  |  |  |  |  |  |  |  |  |  |  |  |  |  | DOT180-2 |  | T | L | - | - | - | - | - |  |
|  |  |  |  |  |  |  |  |  |  |  |  |  |  | DOT180-3 |  | T | L | - | - | - | - | - |  |
|  |  |  |  |  |  |  |  |  |  |  |  |  |  | DOT180-4 |  | T | L | - | - | - | - | - |  |
| Bp_181 | M | 41 | S | 0.8 | - | - | - | WR | 1 | 17/11/24 | D-1 | 2 | Y | DOT181-1 |  | T | N | - | - | - | - | - |  |
|  |  |  |  |  |  |  |  |  |  |  |  |  |  | DOT181-2 |  | T | L | - | - | - | - | - |  |
| Bp_182 | F | 47 | A |  | Y | 4 | 1 | WR | 1 | 18/11/24 | B-14 | 3 | N | DOT182-1 |  | T | L | - | - | - | - | - |  |
|  |  |  |  |  |  |  |  |  |  |  |  |  |  | DOT182-2 |  | T | N | - | - | - | - | - |  |
| Bp_184 | F | 40 | A | - | Y | 4 | 1.4 | WR | 1 | 18/11/24 | C-25 | 1 | N | DOT184-1 | B8988 | T | L | - | - | I.t. | N | N |  |
| Bp_185 | M | 41 | A | 1.0 | - | - | - | WR | 1 | 18/11/24 | D-13 | 1 | Y | DOF185-1 |  | F | - | M | A. r. | NM | N | N |  |
| Bp_188 | M | 43 | S | 0.3 | - | - | - | WR | 1 | 19/11/24 | D-3 | 1 | Y | DOT188-1 |  | T | N | - | - | - | - | - |  |
| Bp_190 | M | 41 | A | 0.5 | - | - | - | Pa | 1 | 20/11/24 | A-7 | 5 | Y | DOT190-1 |  | T | L | - | - | - | - | - |  |
|  |  |  |  |  |  |  |  |  |  |  |  |  |  | DOT190-2 |  | T | L | - | - | - | - | - |  |
|  |  |  |  |  |  |  |  |  |  |  |  |  |  | DOT190-3 |  | T | L | - | - | - | - | - |  |
|  |  |  |  |  |  |  |  |  |  |  |  |  |  | DOF190-1 |  | F | - | M | A. r. | NM | N | N |  |
| Bp_199 | F | 35 | S | - | CD | - | - | Pa | 1 | 20/11/24 | B-11 | 0 | Y | DOF199-1 | B8993 | F | - | M | P. h. | NM | N | N |  |
| Bp_205 | M | 42 | A | 0.6 | - | - | - | Pa | 1 | 20/11/24 | C-11 | 1 | N | DOT205-1 |  | T | D | - | - | - | - | - |  |
| Bp_206 | M | 38 | A | 1.2 | - | - | - | Pa | 1 | 20/11/24 | C-14 | 3 | Y | DOT206-1 | B8984 | T | L | - |  | I. sp. cf. t. | N | N |  |
|  |  |  |  |  |  |  |  |  |  |  |  |  |  | DOF206-1 |  | F | - | F | S.s. | Uns. | N | N |  |
| Bp_212 | M | 38 | A | 1 | - | - | - | Pa | 1 | 21/11/24 | B-24 | 3 | N | DOT212-1 |  | T | L | - | - | - | - | - |  |
| Bp_218 | M | 45 | S | 0.8 | - | - | - | BC | 2 | 22/11/24 | BCP-28 | 0 | Y | DOF218-1 |  | F | - | F | A. r. | NM | N | N |  |
|  |  |  |  |  |  |  |  |  |  |  |  |  |  | DOF218-2 | B8991 | F | - | F | A. r. | NM | N | N |  |
| Bp_225 | F | 49 | A | - | Y | 4 | 1.3 | BC | 4 | 24/11/24 | LBC-16 | 1 | N | DOT225-1 |  | T | N | - | - | - | - | - |  |
| Bp_226 | M | 38 | S | 0.3 | - | - | - | BC | 1 | 22/11/24 | BCP-14 | 4 | N | DOT226-1 |  | T | N | - | - | - | - | - |  |
|  |  |  |  |  |  |  |  |  |  |  |  |  |  | DOT226-2 |  | T | L | - | - | - | - | - |  |
|  |  |  |  |  |  |  |  |  |  |  |  |  |  | DOT226-3 |  | T | L | - | - | - | - | - |  |
|  |  |  |  |  |  |  |  |  |  |  |  |  |  | DOT226-4 |  | T | N | - | - | - | - | - |  |
| Bp_228 | M | 34 | S | 1 | - | - | - | BC | 1 | 22/11/24 | BCP-16 | 2 | N | DOT228-1 |  | T | L | - | - | B.p. | N | N |  |
| Bp_229 | M | 43 | S | 0.3 | - | - | - | BC | 1 | 22/11/24 | BCP-22 | 3 | Y | DOT229-1 |  | T | L | - | - | Uns. | N | N |  |
|  |  |  |  |  |  |  |  |  |  |  |  |  |  | DOT229-2 | B8976 | T | L | - | - | I. sp. cf. t. | N | N |  |
|  |  |  |  |  |  |  |  |  |  |  |  |  |  | DOT229-3 |  | T | L | - | - | - | - | - |  |
|  |  |  |  |  |  |  |  |  |  |  |  |  |  | DOT229-4 |  | T | L | - | - | - | - | - |  |
|  |  |  |  |  |  |  |  |  |  |  |  |  |  | DOF229-1 |  | F | - | M | A. r. | NM | N | N |  |
| Bp_230 | F | 46 | A |  | Y | 4 | 0.8 | BC | 1 | 22/11/24 | A6-8 | 0 | Y | DOF230-1 |  | F | - | F | S.s. | Uns. | N | N |  |
| Bp_231 | M | 39 | S | 1 | - | - | - | BC | 1 | 23/11/24 | BCP-9 | 1 | N | DOT231-1 | B8981 | T | D | - | - | I. sp. cf. t. | N | N |  |
|  |  |  |  |  |  |  |  |  | 2 | 24/11/24 | BCP-9 | 1 | Y | DOF231-1 |  | F | - | F | A. r. | NM | N | N |  |
| Bp_232 | F | 50 | A | - | Y | 4 | 1.5 | BC | 1 | 23/11/24 | BCP-15 | 17 | N | DOT232-1 |  | T | L | - | - | B.p. | N | N |  |
|  |  |  |  |  |  |  |  |  |  |  |  |  |  | DOT232-2 | B8977 | T | D | - | - | I. sp. cf. t. | N | N |  |
|  |  |  |  |  |  |  |  |  |  |  |  |  |  | DOT232-3 |  | T | L | - | - | - | - | - |  |
|  |  |  |  |  |  |  |  |  |  |  |  |  |  | DOT232-4 |  | T | L | - | - | - | - | - |  |
|  |  |  |  |  |  |  |  |  |  |  |  |  |  | DOT232-5 |  | T | L | - | - | - | - | - |  |
|  |  |  |  |  |  |  |  |  |  |  |  |  |  | DOT232-6 |  | T | L | - | - | - | - | - |  |
|  |  |  |  |  |  |  |  |  |  |  |  |  |  | DOT232-7 |  | T | L | - | - | - | - | - |  |
|  |  |  |  |  |  |  |  |  |  |  |  |  |  | DOT232-8 |  | T | L | - | - | - | - | - |  |
|  |  |  |  |  |  |  |  |  |  |  |  |  |  | DOT232-9 |  | T | D | - | - | - | - | - |  |
|  |  |  |  |  |  |  |  |  |  |  |  |  |  | DOT232-10 |  | T | L | - | - | - | - | - |  |
| Bp_234 | M | 36 |  | 0.4 | - | - | - | BC | 1 | 23/11/24 | A6-8 | 5 | Y | DOT234-1 |  | T | L | - | - | - | - | - |  |
|  |  |  |  |  |  |  |  |  |  |  |  |  |  | DOT234-2 |  | T | L | - | - | - | - | - |  |
|  |  |  |  |  |  |  |  |  |  |  |  |  |  | DOT234-3 |  | T | D | - | - | - | - | - |  |
|  |  |  |  |  |  |  |  |  |  |  |  |  |  | DOT234-4 |  | T | L | - | - | - | - | - |  |
|  |  |  |  |  |  |  |  |  |  |  |  |  |  | DOF234-1 | B8994 | F | - | F | S.s. | Uns. | N | N |  |
| Bp_235 | M | 37 | S | 0.6 | - | - | - | BC | 1 | 24/11/24 | BCP-26 | 2 | N | DOT235-1 | B8989 | T | N | - | - | I.t. | N | N |  |
| Bp_236 | M | 39 | S | 0.2 | - | - | - | SR | 1 | 21/11/24 | A-3 | 1 | N | DOT236-1 |  | T | N | - | - | - | - | - |  |
|  |  |  |  |  |  |  |  |  | 3 | 23/11/24 | A-8 | 2 | N | DOT236-2 |  | T | L | - | - | - | - | - |  |
|  |  |  |  |  |  |  |  |  |  |  |  |  |  | DOT236-3 |  | T | L | - | - | - | - | - |  |
| Bp_240 | M | 39 | A | 1 | - | - | - | SR | 3 | 23/11/24 | A-27 | 1 | N | DOT240-1 |  | T | L | - | - | - | - | - |  |
|  |  |  |  |  |  |  |  |  |  |  |  |  |  | DOT240-2 |  | T | D | - | - | - | - | - |  |
| Bp_246 | M | 45 | A | 0.5 | - | - | - | SR | 2 | 23/11/24 | A-9 | 2 | N | DOT246-1 |  | T | L | - | - | - | - | - |  |
|  |  |  |  |  |  |  |  |  |  |  |  |  |  | DOT246-2 |  | T | N | - | - | - | - | - |  |
| Bp_250 | F | 43 | A | - | Y | 4 | - | SR | 1 | 22/11/24 | B-13 | 1 | N | DOT250-1 | B8796 | T | A | - | I. cf. t. | - | - | - |  |
| Bp_256 | M | 40 | S | 0.8 | - | - | - | SR | 1 | 23/11/24 | D-16 | 1 | N | DOT256-1 | B8986 | T | L | - | - | I. sp. cf. t. | N | N |  |
| Bp_257 | M | 38 | A | 0.8 |  |  |  | SR | 1 | 24/11/24 | D-15 | 1 | N | DOT257-1 | B8982 | T | L | - | - | I. sp. cf. t. | N | N |  |

**Note:** *Burramys parvus* were trapped and sampled alongside the team that conducts the annual monitoring survey to collect data for a large longitudinal study and so microchip identification numbers could not be released. *Burramys parvus* ID was created to nominate a unique identification to allow individuals to be referred to, without releasing the microchip identification numbers that are part of an ongoing project. **Abbreviations:** cells with **‘-‘ =** not applicable; **Age:** **A**=Adult, **S**= Subadult; Female reproductive details **NC** = unable to be checked, **D4** = dry and four teats, **OG** = orange and greasy fur in pouch, **CD** = clean empty dry (not bred). **Site:** **RC** = Rough Creek, **SRH** = Snow Ridge Hill, **HJ** = Happy Jacks, **CP** = Charlotte Pass, **WR** = Whites River, **Pa** = Paralyser, **BC** = Blue Cow, **SR** = Summit Road. **Observed fleas:** **Y** = yes, **N** = no; Tick Stage: **A** = adult, **L**= larvae, **N**= nymph, **D**= damaged and unable to determine if nymph or larvae. **Flea sex:** **F** = female, **M** = male, **U** = unknown. **Morphological identification:** **S.s.** = *Stephanocircus simsoni* Rothschild, 1905, **A. s.** *= Acanthopsylla scintilla* (Rothschild, 1936), **A. r.** = *Acanthopsylla rothschildi*., **A.** *= Acanthopsylla* sp., **P. h.** = *Pygiopsylla hoplia*, **I. cf. t**. *=* *Ixodes* cf. *tasmani.* **Molecular identification: I. sp. cf. t.** = *Ixodes* sp. cf. tasmani Neumann, 1899, **I.t.** = *Ixodes* tasmani Neumann, 1899*,* **B.p.**= *Burramys parvus*, **Uns.**= Unsuccessful PCR or sequencing*,* **NM** *= no match.* ***Rickettsia* and *Bartonella* result:** **N** = negative, **P** = positive, **S**= suspect.
